# Swan-Neck Deformity in Cerebral Palsy

**Published:** 2017-01-30

**Authors:** Leyu Chiu, Nicholas S. Adams, Paul A. Luce

**Affiliations:** ^a^Michigan State University College of Human Medicine, Grand Rapids, Mich; ^b^Plastic and Reconstructive Surgery Residency, Grand Rapids Medical Education Partners, Grand Rapids, Mich; ^c^Hand Surgery Centre; Grand Rapids, Mich

**Keywords:** swan-neck deformity, cerebral palsy, superficialis sling, extensor mechanism, lateral band

## DESCRIPTION

**Figure 1:**
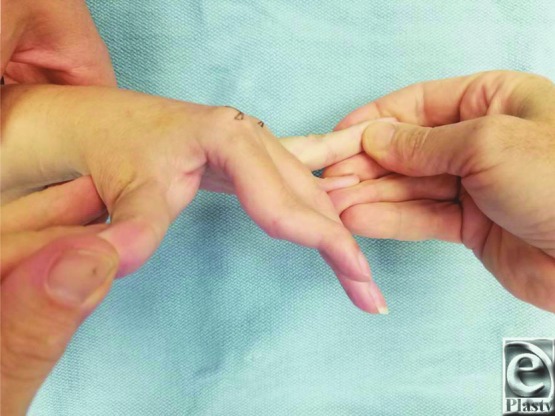
Preoperative view of the left hand demonstrating swan-neck deformity of the index finger.

A 34-year-old woman with cerebral palsy presented with swan-neck deformity of the left index and long fingers (Fig 1). The proximal interphalangeal (PIP) joint was found locked in hyperextension, but the patient could actively flex it if the hyperextension was corrected. The patient was treated with the flexor digitorum superficialis (FDS) sling procedure utilizing suture anchor support.

## QUESTIONS

1. **What are the causes of swan-neck deformity?**
2. **Which major components of the extensor mechanism are involved in swan-neck deformity?**
3. **How are swan-neck deformities in cerebral palsy treated?**
4. **What factors should be taken into consideration in the surgical management of swan-neck deformity?**

## DISCUSSION

Swan-neck deformities are defined by hyperextension of the PIP joint and flexion of the distal interphalangeal (DIP) joint (Fig 1).^1^ The classic disease process associated with swan-neck deformity is rheumatoid arthritis. However, other diseases that weaken the volar plate, such as cerebral palsy, can produce swan-neck deformities. In cerebral palsy, patients can present with swan-neck deformity of 2 different etiologies—intrinsic or extrinsic. The intrinsic type of swan-neck deformity is caused by spastic contraction of the intrinsic muscles of the hand while the extrinsic type is caused by excessive tension of the long extensors due to wrist flexion contractures; these forces lead to weakening of the volar plate of the PIP joint.^2^ Stretching of the volar plate ultimately results in severe hyperextension of the PIP joint that persists after correction of the wrist flexion contractures. In the extrinsic variety, the metacarpophalangeal (MCP) joint remains extended, whereas in the intrinsic variety the MCP joint is contracted into a flexed position. Because of these spastic forces, patients with cerebral palsy often present with advanced swan-neck deformities.

**Figure 2:**
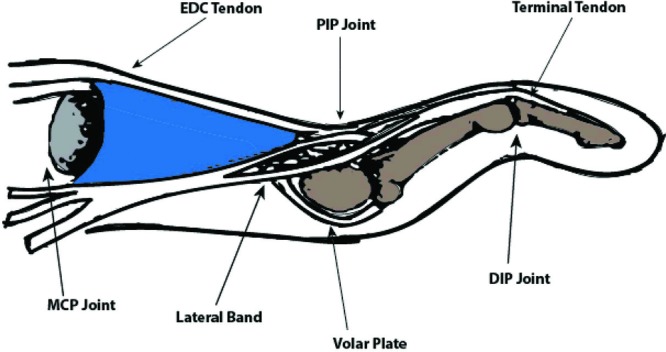
Simplified diagram of the extensor mechanism in swan-neck deformity. Note the dorsal subluxation of the lateral band dorsal to the axis of the PIP joint. Hyperextension leads to attenuation of the volar plate and further hyperextension. DIP indicates distal interphalangeal; EDC, extensor digitorum communis; MCP, metacarpophalangeal; and PIP, proximal interphalangeal.

**Figure 3:**
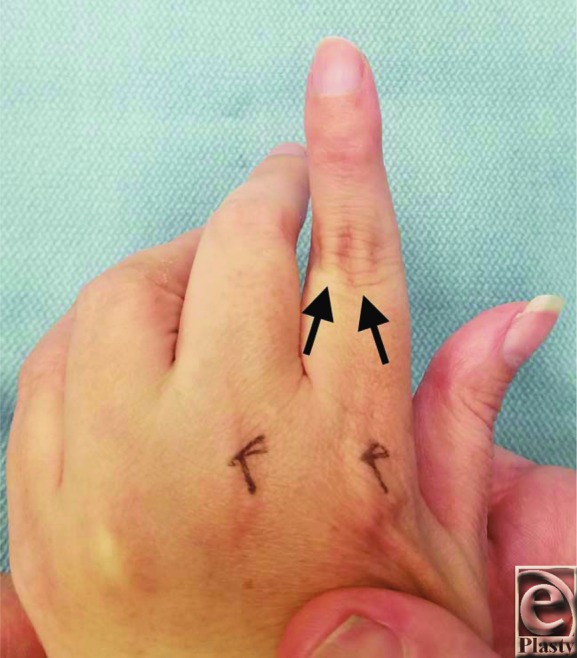
Dorsal view of the left index finger. Attenuation of the transverse retinacular ligaments leads to dorsal translocation of the lateral bands that can be palpated and visualized over the dorsal proximal interphalangeal joint (black arrows).

Flexion and extension of the digital joints are a complex balance between the extrinsic flexors and extensors with the intrinsic muscles of the hand, including the interosseous and lumbrical muscles. Imbalance of these forces leads to stretching and attenuation of the soft-tissue stabilizers of the extensor mechanism. Neurological diseases such as cerebral palsy can cause chronic spasticity of the extrinsic extensors, leading to hyperextension at the PIP joint (Fig 2).^3^ Simultaneously, the volar plate beneath the PIP joint is stretched, leading to decreased resilience and loss of elasticity over time. In addition, swan-neck deformity often results in dorsal subluxation of the lateral bands (Figs 2 and 3). This translocation is caused by attenuation of the transverse retinacular ligaments, the primary retaining ligaments of the lateral band at the PIP joint. The combination of these forces leads to dorsal migration of the lateral bands in relation to the PIP joint axis of rotation, thereby overexerting an extension moment on the PIP joint and loss of extension at the terminal tendon of the DIP joint.^4^

**Figure 4:**
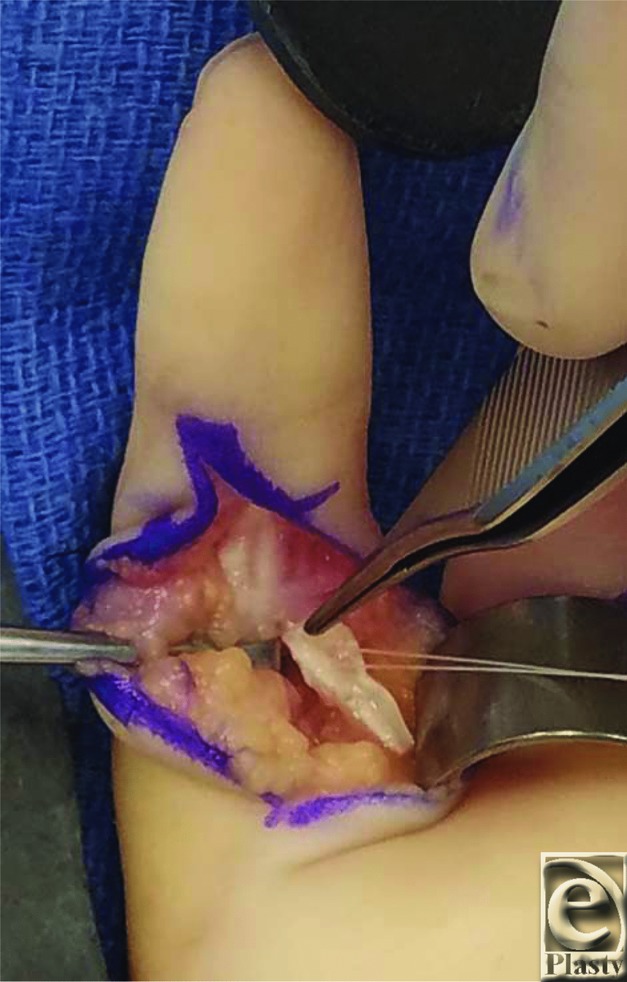
Volar view of the left index finger. The ulnar slip of the flexor digitorum superficialis tendon has been incised proximally and is being secured to the proximal phalanx with a suture anchor. The finger will be maintained in slight flexion while securing the superficialis sling to the proximal phalanx.

There are several treatment options for swan-neck deformities in cerebral palsy. Mild swan-neck deformities are often managed with splinting to prevent locking in hyperextension.^5^ However, this treatment is seldom successful due to the progressive nature of the deformity. Patients who have difficulty initiating flexion and have exhausted nonsurgical options are candidates for surgical treatment, such as the superficialis sling.^5^ The primary goal of the superficialis sling is to provide a volar restraint against hyperextension at the PIP joint.^6^ This surgery has many variations but is often accomplished by isolating one slip of the FDS tendon distal to Camper's chiasm, incising it proximally, slipping it through the A2 pulley, and suturing it onto itself. Additional techniques utilize suture anchors to secure the proximal tendon (Fig 4). The result of this tenodesis will create resistance to hyperextension at the PIP joint and allows flexion of the digits.

Long-term postural deviations from the immediate postsurgical position must be anticipated in swan-neck deformities treated with the superficialis sling. Although there are ongoing discussions about the optimal angle of PIP joint flexion that should be chosen during tenodesis, some suggest that 20° to 30° of flexion is most appropriate.^7^ Since some degree of tissue relaxation is anticipated, securing the angle of flexion at a greater degree than ultimately desired (Fig 4) can account for this phenomenon and prevent recurrent hyperextension.
